# Adaptive Gene Expression Divergence Inferred from Population Genomics

**DOI:** 10.1371/journal.pgen.0030187

**Published:** 2007-10-26

**Authors:** Alisha K Holloway, Mara K. N Lawniczak, Jason G Mezey, David J Begun, Corbin D Jones

**Affiliations:** 1 Section of Evolution and Ecology, University of California Davis, Davis, California, United States of America; 2 Center for Population Biology, University of California Davis, Davis, California, United States of America; 3 Department of Biology, University College London, London, United Kingdom; 4 Department of Biological Statistics and Computational Biology, Cornell University, Ithaca, New York, United States of America; 5 Department of Biology, University of North Carolina, Chapel Hill, North Carolina, United States of America; 6 Carolina Center for Genome Sciences, University of North Carolina, Chapel Hill, North Carolina, United States of America; North Carolina State University, United States of America

## Abstract

Detailed studies of individual genes have shown that gene expression divergence often results from adaptive evolution of regulatory sequence. Genome-wide analyses, however, have yet to unite patterns of gene expression with polymorphism and divergence to infer population genetic mechanisms underlying expression evolution. Here, we combined genomic expression data—analyzed in a phylogenetic context—with whole genome light-shotgun sequence data from six Drosophila simulans lines and reference sequences from D. melanogaster and D. yakuba. These data allowed us to use molecular population genetics to test for neutral versus adaptive gene expression divergence on a genomic scale. We identified recent and recurrent adaptive evolution along the D. simulans lineage by contrasting sequence polymorphism within D. simulans to divergence from D. melanogaster and D. yakuba. Genes that evolved higher levels of expression in D. simulans have experienced adaptive evolution of the associated 3′ flanking and amino acid sequence. Concomitantly, these genes are also decelerating in their rates of protein evolution, which is in agreement with the finding that highly expressed genes evolve slowly. Interestingly, adaptive evolution in 5′ *cis*-regulatory regions did not correspond strongly with expression evolution. Our results provide a genomic view of the intimate link between selection acting on a phenotype and associated genic evolution.

## Author Summary

Changes in patterns of gene expression likely contribute greatly to phenotypic differences among closely related organisms. However, the evolutionary mechanisms, such as Darwinian selection and random genetic drift, which are underlying differences in patterns of expression, are only now being understood on a genomic level. We combine measurements of gene expression and whole-genome sequence data to investigate the relationship between the forces driving sequence evolution and expression divergence among closely related fruit flies. We find that Darwinian selection acting on regions that may control gene expression is associated with increases in gene expression levels. Investigation of the functional consequences of adaptive evolution on regulating gene expression is clearly warranted. The genetic tools available in Drosophila make functional experiments possible and will shed light on how closely related species have responded to reproductive, pathogenic, and environmental pressures.

## Introduction

Changes in gene expression are governed primarily by the evolution of *cis*-acting elements and *trans*-acting factors. Several single-gene studies have combined data on expression, protein abundance, function, and sequence evolution to make powerful statements about the role of adaptive evolution in effecting phenotypic change [1,2]. These case studies of single genes focused on well-described pathways that were known, a priori, to have remarkable expression differences. As such, they may provide a biased view of the population genetic mechanisms controlling gene expression evolution. Thus, the question remains as to which forces, neutral or adaptive, predominate on a genomic level to bring about changes in gene expression.

Recent studies have tried to discern the causes of genome-wide expression evolution solely from patterns of gene expression variation within and among species [3–5]. Patterns of constant expression levels across several species combined with significantly elevated or reduced expression in a single species have been taken as evidence of lineage-specific adaptive evolution [3,4]. Alternatively, low levels of within-population variation in expression compared to divergence in expression among species has also been taken as evidence of adaptive evolution [5–7]. As these studies are based strictly on phenotypic data—expression variation—they are indirect indicators of the underlying genetic and population genetic phenomena. For example, elevated lineage-specific expression divergence can be explained equally well by directional selection or by reduced functional constraint. These studies highlight the importance of direct tests of the mechanisms of evolution. For example, Good et al. [8] used statistical inferences of adaptive protein evolution along with expression evolution to investigate the connection between the two. Their highly conservative test suggested that no significant connection existed. In an attempt to unite population genetic inference with expression data, Khaitovich et al. [9] found a positive correlation between linkage disequilibrium and expression divergence in genes expressed in the human brain. This result is consistent with recent adaptive evolution of *cis*-acting regulatory elements associated with brain-expressed genes, but could also be due to selection on protein function.

A global understanding of the population genetic processes acting on expression phenotypes requires both genomic expression data and genomic sequence variation and divergence data. Combining these data allows for the use of molecular population genetic tests to identify the underlying evolutionary mechanism. To this end, we combined expression data from three closely related species, D. simulans, D. melanogaster, and D. yakuba [6,10], with population genomic sequence data from D. simulans [11], and genome sequence data from D. melanogaster [12] and D. yakuba [11]. These data allow us to polarize both expression and sequence evolution to particular lineages. Additionally, we used the sequence data to mask expression probes (which were developed using the D. melanogaster reference) with sequence mismatches in D. simulans and *D. yakuba*. This approach has the critical advantage that it does not confound expression divergence with sequence evolution across lineages.

DNA polymorphism and divergence data allow one to directly test for both recent and recurrent directional selection on genes and noncoding regions associated with rapid changes in expression. If expression evolution were due to recent directional selection on *cis*-acting elements, we predict a reduction in the DNA heterozygosity to divergence ratio in flanking regions of genes showing expression evolution relative to genomic averages [13]. Alternatively, if recurrent directional selection has acted on *cis*-regulatory sequences controlling expression levels, one might observe excess fixations at regulatory sites relative to nearby “neutrally” evolving sites [14]. Finally, if gene expression diverges primarily due to *trans-*acting factors or neutral processes at *cis*-acting sites, one would expect no evidence of directional selection on noncoding sequences near genes showing expression divergence.

Here, we use population genomic and gene expression data from *Drosophila* to address the following questions: Is expression evolution associated with adaptive evolution of *cis* regions? Are genes with modified expression patterns also evolving modified protein function under directional selection? Are genes that change expression over short time scales clustered into distinct functional groups?

## Results/Discussion

### Expression Analysis

We reanalyzed previously collected expression data from adult male *D. melanogaster, D. simulans,* and D. yakuba from the *Drosophila* v1 Affymetrix GeneChip Array [6,10]. Sequence divergence of probe targets in D. simulans and D. yakuba could confound expression analysis [15], so mismatched probes were masked before analysis. After masking procedures, 4,427 probe sets remained, with an average of 3.81 (SE ± 1.01) probes per set. We defined genes that are increasing and decreasing in expression in D. simulans as those in the 5% tails of expression divergence from the D. melanogaster–D. simulans ancestor (see Materials and Methods).

### Adaptive 3′ *cis*-Regulatory Evolution Associated with Expression Divergence

*Cis-*regulatory element evolution directly affects transcription and mRNA half-life (see [16,17]). *Cis*-acting elements, such as core promoters, that regulate transcription are predominantly located in 5′ regions and those that control mRNA stability and degradation are primarily located in 3′ regions [16,17], although there is considerable variation among genes. We tested for evidence of an association between recent and recurrent directional selection in 5′ and 3′ flanking regions (which include UTRs and putative regulatory regions) and significant changes in expression levels.

**Figure 1:**
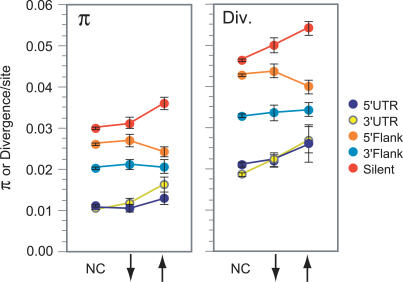
D. simulans Heterozygosity (Left) and Divergence (Right) for Genes with and without Gene Expression Divergence Divergent gene expression is associated with rapid evolution of protein coding and regulatory regions. There is no relationship between heterozygosity and expression divergence. Points are means with standard error. See Table S2 for sample sizes. NC, no change; ↑, increase in expression along the D. simulans lineage; ↓, decrease in expression along the D. simulans lineage.

**Figure 2:**
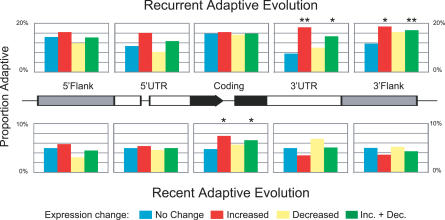
Proportion of Genes Evolving Adaptively in Each Expression Category and for Each Feature Genes with increased expression levels have associated recurrent adaptive evolution of 3′ UTR and 3′ flanking regions as well as evidence for recent adaptive evolution of protein coding regions. For resampling tests, *p < 0.05 and **p < 0.01. Descriptions of tests for recurrent and recent adaptive evolution can be found in the Materials and Methods section.

Reductions in polymorphism relative to divergence indicate the action of recent directional selection [13]. Flanking regions with polymorphism to divergence ratios in the lowest 5% tail of the distribution were taken as having evidence of recent selective sweeps. Figure 1 depicts mean levels of polymorphism and divergence in 5′ and 3′ noncoding sequence. Flanking regions and UTRs have lower levels of polymorphism and divergence than silent sites, which is in agreement with previous findings that noncoding regions are under greater constraint than silent sites [13]. Genes with increased expression levels show more variability in levels of polymorphism and divergence over different features, but no strong pattern emerges. There is no evidence of hitchhiking effects in either 5′ or 3′ UTR or flanking regions in association with changes in expression (Figure 2; Table S1).

Using an extension of the McDonald-Kreitman test [14] for noncoding sites, we compared flanking polymorphic and fixed sites to synonymous sites of the corresponding gene to infer the action of recurrent directional selection. Genes with significant expression evolution show more evidence of recurrent directional selection in 3′ UTRs and 3′ flanking regions than expected by chance (Figure 2; Table S1). Genes with increases in expression drive this relationship. Although genes with reduced expression have more 3′ UTR and flanking region divergence than genes with no change in expression, the tests provide no strong evidence of recurrent adaptation associated with reduced gene expression (Figure 2; Table S1). The 5′ regulatory regions of genes with increased expression show the same trend, but again the result is not statistically significant (Figure 2; Table S1). Thus, recurrent adaptive evolution of 3′ *cis*-regulatory regions likely plays a critical role in adaptive expression increases.

The 3′ regulatory regions are bound by elements, such as microRNAs, that can stabilize or destabilize mRNA (see [18]). Given the linkage between adaptive evolution of 3′ regulatory regions and expression evolution, we hypothesized that microRNAs may be coevolving with their target genes. We retrieved information on known microRNAs and their targets in D. melanogaster from miRBase [19,20]. We found that those microRNAs that regulate a greater number of genes with changes in expression have faster, but not significantly faster, rates of evolution (Spearman's ρ = 0.2065, *p* = 0.1073). Rapid evolution of microRNAs and adaptive expression divergence associated with 3′ regions strongly motivate in-depth investigation of the 3′ flanking regions to uncover the functional mechanisms for transcriptional regulation of genes with significant expression evolution.

Increases in gene expression were more often associated with adaptive evolution than decreases in expression (Figure 2). This observation does not appear to be due to a bias in analysis of the data because expression changes are normally distributed and there is no correlation between estimated ancestral divergence and change in expression (see Materials and Methods). However, continually increasing expression levels cannot persist over long evolutionary time scales. In fact, expression levels are typically under strong stabilizing selection ([5], and see Materials and Methods). A speculative hypothesis for this observation relies on relaxation of codon bias. Begun et al. [11] documented an accumulation of fixations for unpreferred codons in D. simulans. If these unpreferred codons are slightly deleterious and reduce translational efficiency, regulatory regions may be under directional selection to compensate for this phenomenon by making more transcript available for translation.

### Rapid Protein Evolution Accompanies Rapid Gene Expression Divergence

As seen in previous research [6,8], genes with greater absolute levels of expression divergence evolve faster at the protein level (mean dN ± SE 0.0046 ± 0.0003 and 0.0034 ± 0.0001, for genes changing in expression and not changing, respectively; Wilcoxon: *p* < 0.0001; Table S2). Genes with rapid expression evolution are also represented by fewer expression probes per set (mean number of probes ± SE 2.98 ± 0.076 versus 3.90 ± 0.033; Wilcoxon: *p* < 0.0001). A rapid rate of sequence evolution would lead to more probe mismatch, which explains the observed pattern. This also renders our expression divergence analysis conservative, as our power to detect a significant expression difference is reduced for the most rapidly evolving genes. Interestingly, even though genes with significant increases in expression in D. simulans have higher average dN, they show *decelerating* dN in D. simulans relative to D. melanogaster and D. yakuba (resampling test: *p* = 0.023; method for relative rates described in Begun et al. [11]). The same is not true of genes with decreasing expression (*p* = 0.861). While higher average rates of amino acid evolution in genes with expression divergence could have been indicative of relaxed purifying selection, the deceleration in dN certainly speaks against that hypothesis. Previous work showed that high levels of expression correlate with lower rates of protein evolution [21–23], which may reflect selection for translational robustness [23] or translational accuracy [22]. The deceleration in protein evolution of genes with increases in expression is consistent with the idea of stronger translational selection on highly expressed genes, but overall, we see only a weak relationship between expression level and protein divergence (Spearman's ρ = −0.1821, *p* < 0.0001).

### Coding Sequence Evolution Associated with Expression Divergence

Genes adaptively evolving modified expression patterns may also be adaptively evolving modified protein function. We estimated the proportion of genes in each expression class—increasing, decreasing, and no change—with evidence for recurrent directional selection using the McDonald-Kreitman test [14]. For all genes in this analysis, the proportion undergoing recurrent adaptive evolution was similar to the genome-wide estimate [11]. The prevalence of recurrent adaptive evolution was not significantly different for genes showing expression evolution versus those showing no expression evolution (*p* = 0.4438; Figure 2 and Table S1).

We also tested for evidence of recent directional selection as measured by a reduction in the ratio of silent polymorphism to silent divergence [13]. Coding regions with ratios in the lowest 5% tail of the distribution were taken to have evidence for recent selective sweeps. A higher proportion of genes showing expression evolution have significantly reduced ratios of silent site polymorphism to divergence, which is consistent with recent selective sweeps (*p* = 0.0445; Figure 2 and Table S1). Genes with increased expression levels explain more of this relationship than genes with decreased expression (increase *p* = 0.0328, decrease *p* = 0.2530), although both sets have greater reductions of silent polymorphism to divergence ratios than genes that are not changing in expression.

The targets of these putative hitchhiking events may have been nearby regulatory regions in an intron or upstream or downstream of the protein coding region. Alternatively, one possible explanation for the association between upregulation and recent selection on coding regions is codon bias. Gene expression is positively correlated with codon bias [22]. Given this association, hitchhiking effects of preferred codons might increase with increasing levels of expression due to stronger selection for translational accuracy [22]. While there is a higher ratio of preferred to unpreferred polymorphisms and fixations in genes evolving increases in expression versus those that show no expression evolution, the difference is not statistically significant (Fisher's Exact Test: *p* ≫ 0.05 for both tests; Table 1). There may be a time lag between expression evolution and the fine-tuning of translation via codon bias. Thus, our data might mean that genes with the most extreme expression differences have recently increased expression. Alternatively, the hitchhiking events may result from adaptive evolution acting on one or a few amino acids or on nearby regulatory regions.

**Table 1:**
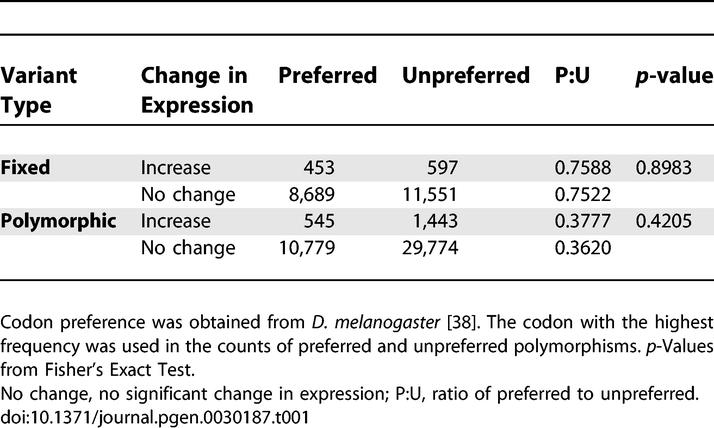
No Evidence for Codon Bias with Increased Expression

### Gene Ontology Analysis

We used gene ontology information from Flybase and from the generic Gene Ontology Slim set of terms to determine whether certain functional classes of genes were more likely to evolve expression differences. Six ontology terms are significantly enriched for genes both with significant increases and decreases in expression (Table S3). Two of those terms, chymotrypsin and trypsin activity, have completely overlapping genes and are part of a larger category, serine-type endopeptidase activity. These genes have many functions, including reproduction, digestion, and immunity [24]. Three other categories, courtship behavior, negative regulation of transcription, and sex determination appear to be unrelated on the surface, but closer inspection of the genes in these categories reveals that all are involved in regulation of transcription or chromatin remodeling. These functions frequently evinced adaptive protein evolution in the genome-wide analysis of adaptive evolution in D. simulans [11]. This suggests that there may be a connection between adaptive protein evolution and expression divergence for some biological functions.

Because adaptive evolution of 3′ *cis*-regulatory regions may be driving expression divergence, at least for genes with increased expression, we examined the classes of genes associated with genes that have both evidence for adaptive 3′ evolution and significant expression divergence (Tables S4 and S5). We also investigated ontology terms associated with genes showing evidence of hitchhiking events and significant expression divergence (Table S6). Generally, genes with adaptive 3′ or protein evolution are found in the cytoplasm or are integral to the membrane. Their molecular functions are predominantly protein binding, nucleic acid binding, and translation related. The most common biological processes are related to response to stimuli, RNA regulation (binding, splicing, degradation), and metabolism.

### Conclusions

In this study, we link adaptive sequence evolution to phenotypic change on a genome-wide scale. Several recent studies have illustrated the importance of adaptive evolution acting on noncoding DNA [11,25,26], and our data reinforce this point. More critically, we show that adaptive evolution of *cis*-acting elements in 3′ regions is clearly associated with and may be driving lineage-specific increases in expression that lead to phenotypic differences among species. Recent work suggests that genes with certain 5′ promoter elements show an increased interspecies *variability* in expression in yeast as well as *Drosophila* [27]. In contrast, our data implies that 3′ regulatory regions are playing a more critical role in adaptive expression divergence. Functional genomic investigation of these 3′ *cis*-regulatory regions is clearly warranted. The question now becomes, how and why do genes involved in important processes such as chromatin remodeling change their expression patterns through 3′ *cis*-acting regulatory adaptive evolution?

## Materials and Methods

### RNA expression data.

We reanalyzed expression data from 3-d-old virgin adult males of one isogenic line of D. melanogaster, ten isogenic lines of D. simulans, and one isogenic line of D. yakuba [6,10]. Three replicate chips for each line were used. All data were collected at the same location under standard conditions using the Affymetrix GeneChip Arrays (*Drosophila* 1.0), which contain 13,966 features representing the genome of *D. melanogaster.* Because the D. melanogaster gene annotation has been updated since the array was developed, we compared probe sequences to the D. melanogaster genome to determine which genes were targeted with each probe set.

### Masking approach.

The probes representing features on the Affymetrix GeneChip Arrays are constructed for D. melanogaster and are not expected to perfectly match other species. Prior research suggests that such imperfect matches cause incorrect measures of expression due to poor hybridization [10,15,28]. To account for the confounding effect of probe sequence divergence among species on gene expression measures, only probes that were identical matches to the genome sequences of D. melanogaster, D. simulans, and D. yakuba were included in analyses. Probes showing any divergence among the probe sequence on the array and the genome sequences of the three species were masked. Probe sets with fewer than two probes remaining after masking (out of the original 14) were removed before downstream analyses. Finally, probe sets that bound to overlapping genes or homologous sequence of multiple genes were also removed, as the signal could not be attributed to a single gene.

### Expression analysis.

After probe-masking procedures, all chips were normalized and expression intensities were calculated using *gcrma* from the *affy* package available in Bioconductor [29,30]. The mean of the log_2_ expression intensity for each probe set was then calculated for each species. Probe sets for which the log_2_ mean intensity of at least one species was not greater than three were considered absent. Of the original 195,944 probes from 13,996 probe sets, 16,850 probes representing 4,427 probe sets remained after masking and removing probe sets with no detectable expression in either D. melanogaster or D. simulans (all expression data are in Table S7). The distribution of expression intensities was highly similar between species (Figure S1) and probe set intensities were highly correlated between species (Spearman's ρ = 0.92 between D. simulans and D. melanogaster and ρ = 0.89 between D. simulans and D. yakuba). However, probe sets with fewer probes have higher coefficients of variation in D. simulans and in D. melanogaster (Kruskal-Wallis tests: *p* < 0.0001 for all four tests). We tested whether probe sets with fewer probes gave reliable estimates of mean expression intensity. We randomly sampled four probes from probe sets that had all 14 probes remaining after masking. The mean expression intensity of the sample was highly correlated with the mean intensity estimated from all 14 probes (Spearman's ρ = 0.869). The mean expression level varied by +/− 7%, and the variance in expression among replicates increased by 22%.

Ancestral expression states were reconstructed using AncML v 1.0 [31] using the average of normalized log_2_ expression values for each species. Expression divergence was calculated as follows:

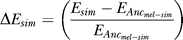
where *E_sim_* is the expression level of D. simulans and *E_Ancmel-sim_* is the estimated expression level of the D. simulans/*melanogaster* ancestor. Figure S2 depicts the distribution of expression change along the D. simulans lineage. The distribution is not significantly different from normally distributed. Additionally, there is no correlation between change in expression along the D. simulans branch and the expression level of the inferred ancestor (Figure S3). The conical nature of Figure S3 reflects the negative correlation between expression level and expression divergence over short evolutionary time scales. We defined genes that are increasing and decreasing in expression in D. simulans as those in the 5% tails of expression divergence from the *D. melanogaster–D. simulans* ancestor. We calculated confidence intervals (CI) around the expression values for D. simulans and determined whether the D. melanogaster expression estimate fell within the D. simulans CI. Intraspecific expression divergence values in the tails are not normally distributed, so we calculated CIs in R using bias correction and acceleration [32]. One probe set (of 221) with increasing expression and four probe sets (of 221) with decreasing expression along the D. simulans lineage had mean intensities in D. melanogaster within the 95% CIs of D. simulans.

### Analysis of syntenic assembly.

Drosophila simulans and D. yakuba syntenic assemblies are described in Begun et al. [11] and information on the D. yakuba genome project can be found at http://genome.wustl.edu. From light-shotgun sequencing of six lines of D. simulans, a total of 109 Mbp of euchromatic sequence were covered by at least one of the six lines. Each line had 43%–90% coverage of that 109 Mbp with an average of 3.6 alleles per site. However, coverage of genic regions was somewhat higher at 3.9 alleles per site.

Genes and Affymetrix probes were localized using the Flybase v.4.2 annotation (http://flybase.org/annot). Genes included were from two categories. The first set maintained the gene model of D. melanogaster meaning that, in D. simulans, they have canonical translation initiation codons (or that matched the D. melanogaster noncanonical codon), canonical splice junctions at the same position as D. melanogaster (or noncanonical splice junctions that were identical to the D. melanogaster nucleotides at splice sites), no premature termination, and a canonical termination codon. The second set was less conservative in that the gene could have a different gene model with respect to only one of the aforementioned criteria (i.e., either a noncanonical translation initiation codon at the D. melanogaster initiation site, or noncanonical splice junctions, or lack a termination codon at the D. melanogaster termination). Additionally, genes with premature terminations in the last exon were included. There were very few genes with imperfect models in any of the expression groups (10/212 with increased expression, 14/210 with decreased expression, and 173/3,814 with no change in expression). Only gold collection UTRs (i.e., those with completely sequenced cDNAs) were used in analyses (http://www.fruitfly.org/EST/gold_collection.shtml). Flanking regions consisted of sequence 1,000 bases upstream and downstream of any annotated UTR sequence for each gene (or initiation/termination codons for genes without annotated UTRs). Flanking sequence was truncated if the coding sequence of a neighboring gene was within the 1,000 bases. We also investigated 300 bases upstream of the 5′ UTR (see Table S1), which would target core promoter regions, and recovered the same results as with 1,000 bases upstream.

### Statistical tests and parameter estimation.

Some statistical tests were performed using JMP IN v5.1 (SAS Institute). PERL scripts for calculations of estimated nucleotide diversity (π), McDonald-Kreitman tests, and resampling tests were written by and can be obtained from AKH. Nucleotide diversity was estimated as in Begun et al. [11] for each genomic feature (exon, intron, UTRs, flanking) that had a minimum number of nucleotides represented [i.e., *n* (*n* − 1) × *s* ≥ 100, where *n* = average number of alleles sampled and *s* = number of sites]. The measure of nucleotide diversity, π, is the coverage-weighted average expected heterozygosity of nucleotide variants and is therefore an unbiased estimate of polymorphism. For coding regions, the numbers of silent and replacement sites were counted using the method of Nei and Gojobori [33]. The pathway between two codons was calculated as the average number of silent and replacement changes from all possible paths between the pair. Estimates of π on the X chromosome were corrected for sample size [π_ w_ = π × (4/3)] under the assumption that males and females have equal population sizes. Lineage-specific divergence was estimated by maximum likelihood using PAML v3.14 [34] and was reported as a weighted average over each D. simulans line with greater than 50 aligned sites in the segment being analyzed. PAML was run in batch mode using a BioPerl wrapper [35]. For noncoding regions, we used baseml with HKY as the model of evolution to account for transition/transversion bias and unequal base frequencies [36], and for coding regions we used codeml with codon frequencies estimated from the data. For all genes, 0.001 was added to heterozygosity and divergence values so that we could calculate ratios for genes with entries of zero. We did not analyze genes with zero values for both heterozygosity and divergence. Even after correction for smaller effective population sizes, heterozygosity at silent sites is significantly lower on the X chromosome than on autosomes (Kruskal-Wallis test: *p* < 0.0001, Tukey's HSD shows X is different from all autosomes), so we defined significantly low heterozygosity/divergence ratios separately for the X and autosomes. For each feature, genes in the lowest 5% tail of silent site heterozygosity/divergence ratios were defined as being significantly low and therefore showing evidence of a recent selective sweep. Those ratios defined as having evidence of recent selective sweeps were at least 10-fold lower than the mean ratio for all features. D. simulans–specific accelerations/decelerations in protein evolution were calculated as described in Begun et al. [11].

Polarized MK tests minimized the numbers of nonsynonymous substitutions and required that D. melanogaster and D. yakuba share the same codon to ensure that fixations and polymorphisms were attributable to evolution along the D. simulans lineage. We used a derivative of the McDonald-Kreitman test [14] to evaluate evidence for recurrent directional selection in noncoding regions. Polymorphic and fixed sites of noncoding DNA were compared to polymorphic and fixed silent sites of the gene. Again, we only analyzed sites where D. melanogaster and D. yakuba shared the same nucleotide.

With very few polymorphisms and fixations there is little power to detect the action of directional selection. Therefore, we imposed a minimum row and column count for tests to be included in downstream analyses. We required that each row and column in the 2 × 2 table have a sum of at least five observations. We also removed any tests that had a significant test result but that had a neutrality index value greater than one, (which indicates excess amino acid/noncoding polymorphism not directional selection [37]) in order to calculate the proportion of genes that are experiencing recurrent directional selection. All data for D. simulans heterozygosity, lineage-specific divergence and MK tests are listed in Table S8. Substitutions to preferred and unpreferred codons were estimated by a parsimony method developed by Y.-P. Poh [11].

### Resampling tests.

For each category of interest (e.g., increasing or decreasing expression levels), we calculated the proportion of genes with a significant test result (for MK tests, *p* ≤ 0.05, for heterozygosity/divergence ratios were considered significant if they fell in the 5% tail). We then tested whether this proportion was significantly greater than the random expectation using resampling tests. We randomly drew *n p*-values from the set of all genes where *n* is the number of genes in the category. We repeated this procedure 10,000 times to get the empirical distribution of proportion genes with significant tests.

### Gene ontology.

We obtained cellular component, molecular function, and biological process ontology terms from the Flybase gene ontology terms (http://flybase.org/genes/lk/function) in combination with the generic Gene Ontology Slim set of ontology terms (http://geneontology.org/GO.slims.shtml#avail). The proportion of genes with significant expression evolution was calculated for each ontology term. We determined whether each ontology term had a higher proportion of genes with significant D. simulans expression divergence than would be expected from the empirical distribution. We derived the empirical distribution for each ontology term by drawing the same number of genes as was in the term from all genes with expression data. We then calculated the proportion in the resampled dataset with significant expression evolution. We used 10,000 resampled data sets to derive the empirical distribution for each term.

## Supporting Information

Figure S1 Distribution of Expression Intensities in D. melanogaster, D. simulans, and D. yakuba
(60 KB DOC)

Figure S2 Distribution of Expression Divergence along the D. simulans Lineage (562 KB DOC)

Figure S3 Relationship between Change in Expression and Estimated Ancestral Expression Levels (73 KB DOC)

Table S1 Recurrent and Recent Selection on Coding Regions and *cis*-Regulatory Regions (99 KB DOC)

Table S2 D. simulans Heterozygosity and Lineage-Specific Divergence for Protein Coding and *cis*-Regulatory Regions (60 KB DOC)

Table S3 Ontology Categories with Enrichment of Genes with Both Significant Increases and Decreases in Expression (82 KB DOC)

Table S4 Gene Ontology Information for Gene with Increases in Expression and Evidence for Adaptive Evolution in the 3′ UTR (126 KB DOC)

Table S5 Gene Ontology Information for Gene with Increases in Expression and Evidence for Adaptive Evolution in 3′ Flanking Regions (234 KB DOC)

Table S6 Gene Ontology Information for Gene with Increases in Expression and Evidence for Recent Adaptive Evolution in the Coding Region (192 KB DOC)

Table S7 Gene Expression Data (1.4 MB XLS)

Table S8 Heterozygosity, Divergence, and Counts of Polymorphic and Fixed Sites for Each Feature (3.4 MB XLS)

## Abbreviations

CI: confidence interval
