# Supplementary figures and images for: Adaptive Gene Expression Divergence Inferred from Population Genomics
Source: PLoS Genet. 2007 Oct 26;3(10):e187. doi: 10.1371/journal.pgen.0030187 (PMC2042001; doi:10.1371/journal.pgen.0030187)

Figure S1. Distribution of expression intensities in *D. melanogaster*, *D. simulans*, and *D. yakuba*.


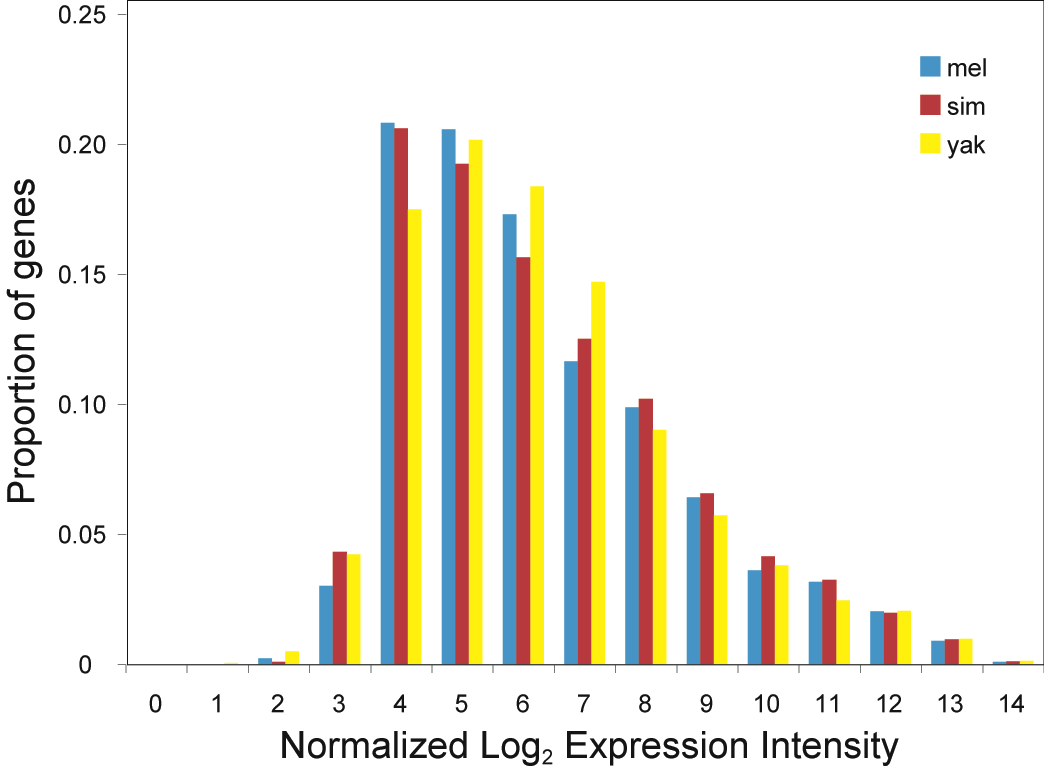

Supplement: Figure S1 — (60 KB DOC) [file pgen.0030187.sg001.doc]

Figure S2. Distribution of expression divergence along the *D. simulans* lineage.


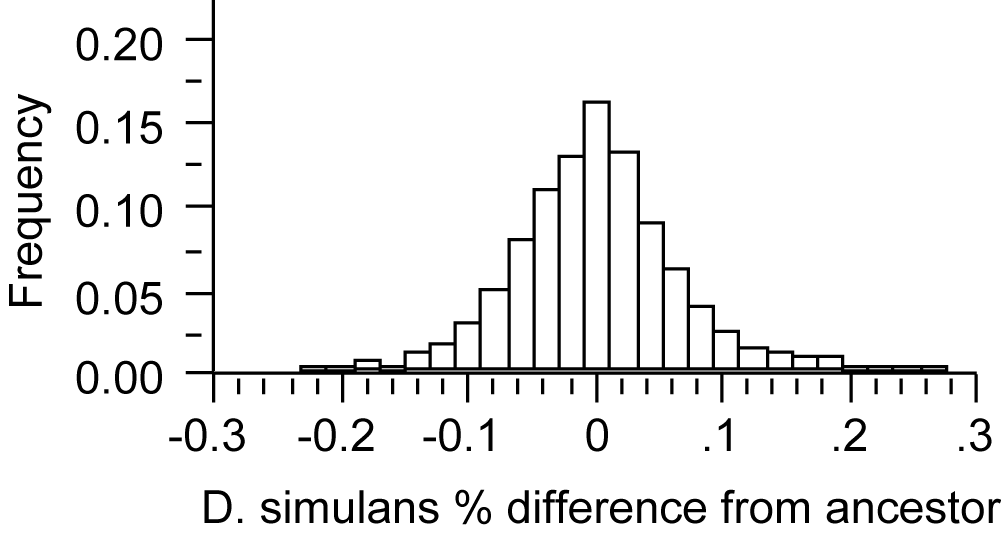

Supplement: Figure S2 — (562 KB DOC) [file pgen.0030187.sg002.doc]
