# Supplementary material for: Adaptive Gene Expression Divergence Inferred from Population Genomics
Source: PLoS Genet. 2007 Oct 26;3(10):e187. doi: 10.1371/journal.pgen.0030187 (PMC2042001; doi:10.1371/journal.pgen.0030187)

Figure S3. Relationship between change in expression and estimated ancestral expression levels.


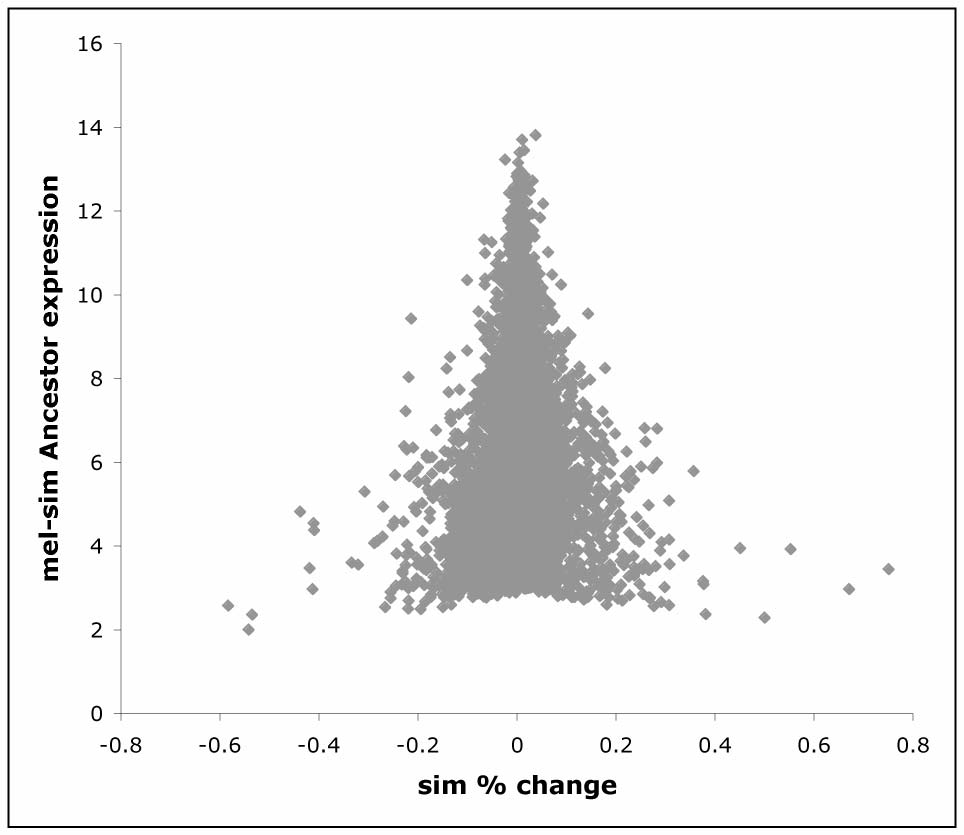

Supplement: Figure S3 — (73 KB DOC) [file pgen.0030187.sg003.doc]
