# Supplementary material for: Adaptive Gene Expression Divergence Inferred from Population Genomics
Source: PLoS Genet. 2007 Oct 26;3(10):e187. doi: 10.1371/journal.pgen.0030187 (PMC2042001; doi:10.1371/journal.pgen.0030187)
Supplement: Table S1 — (99 KB DOC) [file pgen.0030187.st001.doc]

Table S1. Recurrent and recent selection on coding regions and *cis*-regulatory regions.

| Region | Expression  Evolution^†^ | Recurrent Selection | | |  | Recent Selection | | |
| --- | --- | --- | --- | --- | --- | --- | --- | --- |
|  |  | No. genes | Prop. p<=0.05 | p-value |  | No. genes | Prop. Low 5% | p-value |
| 5’UTR | nc | 560 | 0.107 |  |  | 1397 | 0.050 |  |
|  | ↑ | 31 | 0.161 | 0.1063 |  | 74 | 0.054 | 0.3155 |
|  | ↓ | 24 | 0.083 | 0.4886 |  | 65 | 0.046 | 0.4144 |
|  | ↑or↓ | 55 | 0.127 | 0.2385 |  | 139 | 0.050 | 0.3931 |
|  |  |  |  |  |  |  |  |  |
| 3’UTR | nc | 734 | 0.076 |  |  | 1557 | 0.050 |  |
|  | ↑ | 38 | 0.184 | ****0.0093** |  | 86 | 0.035 | 0.6376 |
|  | ↓ | 30 | 0.100 | 0.2299 |  | 72 | 0.069 | 0.1464 |
|  | ↑or↓ | 68 | 0.147 | ***0.0174** |  | 158 | 0.051 | 0.3984 |
|  |  |  |  |  |  |  |  |  |
| 5’Flank | nc | 2626 | 0.144 |  |  | 3299 | 0.050 |  |
|  | ↑ | 140 | 0.164 | 0.1984 |  | 171 | 0.058 | 0.2361 |
|  | ↓ | 135 | 0.119 | 0.7602 |  | 160 | 0.031 | 0.8203 |
|  | ↑or↓ | 275 | 0.142 | 0.4840 |  | 331 | 0.045 | 0.5947 |
|  |  |  |  |  |  |  |  |  |
| 5’Flank300^‡^ | nc | 2204 | 0.126 |  |  | 3260 | 0.051 |  |
|  | ↑ | 121 | 0.149 | 0.1737 |  | 168 | 0.060 | 0.2194 |
|  | ↓ | 114 | 0.105 | 0.6947 |  | 158 | 0.032 | 0.8120 |
|  | ↑or↓ | 235 | 0.126 | 0.4265 |  | 326 | 0.046 | 0.5851 |
|  |  |  |  |  |  |  |  |  |
| 3’Flank^‡^ | nc | 1956 | 0.117 |  |  | 3151 | 0.050 |  |
|  | ↑ | 105 | 0.181 | ***0.0225** |  | 168 | 0.036 | 0.7423 |
|  | ↓ | 105 | 0.143 | 0.1922 |  | 154 | 0.052 | 0.3605 |
|  | ↑or↓ | 210 | 0.162 | ***0.0246** |  | 322 | 0.043 | 0.6532 |
|  |  |  |  |  |  |  |  |  |
| Coding | nc | 1070 | 0.158 |  |  | 3827 | 0.048 |  |
|  | ↑ | 67 | 0.164 | 0.3649 |  | 213 | 0.075 | ***0.0328** |
|  | ↓ | 72 | 0.153 | 0.4719 |  | 210 | 0.057 | 0.2530 |
|  | ↑or↓ | 139 | 0.158 | 0.4438 |  | 423 | 0.066 | ***0.0445** |
|  |  |  |  |  |  |  |  |  |
| Intron1^§^ | nc | 1828 | 0.120 |  |  | 3188 | 0.050 |  |
|  | ↑ | 101 | 0.109 | 0.5518 |  | 172 | 0.058 | 0.2425 |
|  | ↓ | 96 | 0.083 | 0.8125 |  | 163 | 0.049 | 0.4338 |
|  | ↑or↓ | 199 | 0.096 | 0.8152 |  | 335 | 0.054 | 0.3131 |

^†^nc = no significant change in expression; ↑ = increase in expression; ↓ = decrease in expression.

^‡^We analyzed 300 bp 5’ of each UTR, which would target the core promoter region. (see Ohler, U., Liao, G.C., Niemann, H. & Rubin, G.M. 2002. Computational analysis of core promoters in the Drosophila genome. *Genome Biol* **3**, RESEARCH0087)

^§^We analyzed the 1^st^ intron of each gene. As in coding regions pi/divergence ratios were lower for genes with increases in expression, but were not significantly different from the empirical distribution.

* p <= 0.05, ** p <= 0.01.
