# Supplementary material for: Adaptive Gene Expression Divergence Inferred from Population Genomics
Source: PLoS Genet. 2007 Oct 26;3(10):e187. doi: 10.1371/journal.pgen.0030187 (PMC2042001; doi:10.1371/journal.pgen.0030187)
Supplement: Table S2 — (60 KB DOC) [file pgen.0030187.st002.doc]

Table S2. *D. simulans* heterozygosity and lineage-specific divergence for protein coding and *cis*-regulatory regions.

| Feature | Expression  Change^*^ | N | π^†^ | ML divergence^†^ |
| --- | --- | --- | --- | --- |
| 5’UTR | nc | 1397 | 0.0109 (0.0003) | 0.0209 (0.0005) |
|  | ↑ | 74 | 0.0104 (0.0083) | 0.0217 (0.0016) |
|  | ↓ | 65 | 0.0128 (0.0015) | 0.0261 (0.0045) |
|  |  |  |  |  |
| 3’UTR | nc | 1557 | 0.0104 (0.0003) | 0.0187 (0.0005) |
|  | ↑ | 86 | 0.0115 (0.0011) | 0.0223 (0.0016) |
|  | ↓ | 72 | 0.0160 (0.0018) | 0.0271 (0.0032) |
|  |  |  |  |  |
| ’Flank | nc | 3299 | 0.0258 (0.0003) | 0.0427 (0.0004) |
|  | ↑ | 171 | 0.0267 (0.0014) | 0.0438 (0.0016) |
|  | ↓ | 160 | 0.0238 (0.0012) | 0.0400 (0.0016) |
|  |  |  |  |  |
| 3’Flank | nc | 3151 | 0.0199 (0.0003) | 0.0329 (0.0005) |
|  | ↑ | 168 | 0.0209 (0.0013) | 0.0336 (0.0019) |
|  | ↓ | 154 | 0.0204 (0.0015) | 0.0343 (0.0017) |
|  |  |  |  |  |
| Synonymous | nc | 3827 | 0.0297 (0.0003) | 0.0464 (0.0004) |
|  | ↑ | 213 | 0.0311 (0.0013) | 0.0501 (0.0017) |
|  | ↓ | 210 | 0.0357 (0.0016) | 0.0541 (0.0017) |

^*^nc = no significant change in expression; ↑ = increase in expression; ↓ = decrease in expression.

^†^Mean (SE).
