# Supplementary material for: Adaptive Gene Expression Divergence Inferred from Population Genomics
Source: PLoS Genet. 2007 Oct 26;3(10):e187. doi: 10.1371/journal.pgen.0030187 (PMC2042001; doi:10.1371/journal.pgen.0030187)
Supplement: Table S3 — (82 KB DOC) [file pgen.0030187.st003.doc]

Table S3. Ontology categories with enrichment of genes with both significant increases and decreases in expression.

| GOID | Computed Gene ID | Gene Name | | Major Functions |
| --- | --- | --- | --- | --- |
| GO:0007619 – courtship behavior | | | | |
|  | CG12390 | dare | | olfaction, electron transporter activity |
|  | CG7925 | technical knockout (tko)* | | male courtship, sense sound |
|  | CG11094 | doublesex (dsx) | | reg. transc., reproduction related |
|  | CG17228 | prospero (pros) | | reg. transc., sense taste, develop. |
|  |  |  | |  |
| GO:0007530 – sex determination | | | | |
|  | CG6118 |  | | chrom. assembly/dis., reg. transc. |
|  | CG3496 | virilizer (vir)* | | reg. splicing, dosage comp. |
|  | CG9401 | mago | | microtubule associated |
|  | CG11094 | doublesex (dsx) | | reg. transc., reproduction related |
|  | CG14307 | fruitless (fru) | | reg. transc., sex/courtship |
|  |  |  | |  |
| GO:0045892 – negative regulation of transcription | | |  | |
|  | CG8409 | Su(var)205 | | chrom. silencing |
|  | CG8411 | gcl | | nuclear pore protein |
|  | CG11094 | doublesex (dsx) | | reg. transc., reproduction related |
|  | CG4013 | Smr | |  |
|  |  |  | |  |
| GO:0019207 – kinase regulator activity | | |  | |
|  | CG5212 | pellino(Pli) | |  |
|  | CG9031 | Ras suppressor-1 (Rsu-1) | |  |
|  | CG18292 |  | |  |
|  | CG4946 |  | |  |
|  |  |  | |  |
| GO:0004263 – chymotrypsin activity  GO:0004295 – trypsin activity | | |  | |
|  | CG7142 | SP132 | |  |
|  | CG7829 | SP136 | |  |
|  | CG8172 | SP32 | |  |
|  | CG8299 | SP65 | |  |
|  | CG9564 | Try29F | |  |
|  | CG10475 | Jon65Ai* | |  |
|  | CG11836 | SP23 | |  |
|  | CG17571 | SP83 | |  |
|  | CG18180 | SP151 | |  |
