# Supplementary material for: Adaptive Gene Expression Divergence Inferred from Population Genomics
Source: PLoS Genet. 2007 Oct 26;3(10):e187. doi: 10.1371/journal.pgen.0030187 (PMC2042001; doi:10.1371/journal.pgen.0030187)
Supplement: Table S4 — (126 KB DOC) [file pgen.0030187.st004.doc]

Table S4. Gene Ontology information for gene with increases in expression and evidence for adaptive evolution in the 3’UTR.

| Gene | Cellular Component | | Molecular Function | | Biological Process | |
| --- | --- | --- | --- | --- | --- | --- |
| blistery (CG9379) | GO:0005623 | cell | GO:0003779 | actin binding | GO:0007475 | apposition of dorsal and ventral wing surfaces |
|  | GO:0005925 | focal adhesion | GO:0005488 | binding | GO:0016043 | cell organization and biogenesis |
|  | GO:0005886 | plasma membrane | GO:0008092 | cytoskeletal protein binding | GO:0050875 | cellular physiological process |
|  |  |  | GO:0005515 | protein binding | GO:0007028 | cytoplasm organization and biogenesis |
|  |  |  | GO:0005200 | structural constituent of cytoskeleton | GO:0007016 | cytoskeletal anchoring |
|  |  |  | GO:0005198 | structural molecule activity | GO:0007010 | cytoskeleton organization and biogenesis |
|  |  |  |  |  | GO:0007275 | development |
|  |  |  |  |  | GO:0007242 | intracellular signaling cascade |
|  |  |  |  |  | GO:0007498 | mesoderm development |
|  |  |  |  |  | GO:0009653 | morphogenesis |
|  |  |  |  |  | GO:0007517 | muscle development |
|  |  |  |  |  | GO:0006996 | organelle organization and biogenesis |
|  |  |  |  |  | GO:0007582 | physiological process |
|  |  |  |  |  | GO:0007476 | wing morphogenesis |
|  |  |  |  |  |  |  |
| Cyp4p2 (CG1944) | GO:0005623 | cell | GO:0003824 | catalytic activity | GO:0006118 | electron transport |
|  | GO:0016020 | membrane | GO:0004497 | monooxygenase activity | GO:0006629 | lipid metabolism |
|  | GO:0005792 | microsome | GO:0016491 | oxidoreductase activity | GO:0008152 | metabolism |
|  |  |  | GO:0005215 | transporter activity | GO:0007582 | physiological process |
|  |  |  |  |  | GO:0008202 | steroid metabolism |
|  |  |  |  |  |  |  |
| Gclc (CG2259) | GO:0005623 | cell | GO:0005488 | binding | GO:0006519 | amino acid and derivative metabolism |
|  | GO:0005737 | cytoplasm | GO:0003824 | catalytic activity | GO:0006520 | amino acid metabolism |
|  | GO:0017109 | glutamate-cysteine ligase complex | GO:0004357 | glutamate-cysteine ligase activity | GO:0009058 | biosynthesis |
|  | GO:0005622 | intracellular | GO:0003676 | nucleic acid binding | GO:0006750 | glutathione biosynthesis |
|  |  |  | GO:0005515 | protein binding | GO:0008152 | metabolism |
|  |  |  |  |  |  |  |
| Table 4 continued. | | | | | | |

| Gene | Cellular Component | Molecular Function | Biological Process |
| --- | --- | --- | --- |

| Gclc cont. |  |  |  |  | GO:0007582 | physiological process |
| --- | --- | --- | --- | --- | --- | --- |
|  |  |  |  |  | GO:0006412 | protein biosynthesis |
|  |  |  |  |  | GO:0019538 | protein metabolism |
|  |  |  |  |  |  |  |
| CG10475 |  |  | GO:0003824 | catalytic activity | GO:0009056 | catabolism |
| Jon65Ai |  |  | GO:0004263 | chymotrypsin activity | GO:0008152 | metabolism |
|  |  |  | GO:0016787 | hydrolase activity | GO:0007582 | physiological process |
|  |  |  | GO:0008233 | peptidase activity | GO:0019538 | protein metabolism |
|  |  |  | GO:0004252 | serine-type endopeptidase activity | GO:0006508 | proteolysis |
|  |  |  | GO:0004295 | trypsin activity |  |  |
|  |  |  |  |  |  |  |
| CG9842 | GO:0005955 | calcineurin complex | GO:0005488 | binding | GO:0008152 | metabolism |
| Pp2B-14D |  |  | GO:0004723 | calcium-dependent protein serine/threonine phosphatase activity | GO:0007582 | physiological process |
|  |  |  | GO:0005516 | calmodulin binding | GO:0006470 | protein amino acid dephosphorylation |
|  |  |  | GO:0003824 | catalytic activity | GO:0019538 | protein metabolism |
|  |  |  | GO:0016787 | hydrolase activity | GO:0006464 | protein modification |
|  |  |  | GO:0004721 | phosphoprotein phosphatase activity |  |  |
|  |  |  | GO:0005515 | protein binding |  |  |
|  |  |  | GO:0004722 | protein serine/threonine phosphatase activity |  |  |
