# Supplementary material for: Adaptive Gene Expression Divergence Inferred from Population Genomics
Source: PLoS Genet. 2007 Oct 26;3(10):e187. doi: 10.1371/journal.pgen.0030187 (PMC2042001; doi:10.1371/journal.pgen.0030187)
Supplement: Table S5 — (234 KB DOC) [file pgen.0030187.st005.doc]

Table S5. Gene Ontology information for gene with increases in expression and evidence for adaptive evolution in 3’flanking regions.

| Gene | Cellular Component | Molecular Function | Biological Process |
| --- | --- | --- | --- |
| alpha-Esterase-4 (CG1082) |  | carboxylesterase activity |  |
|  |  | catalytic activity |  |
|  |  | hydrolase activity |  |
|  |  |  |  |
| CG3960 | integral to membrane | transporter activity | cell communication |
|  |  |  | cell-cell signaling |
|  |  |  | neurotransmitter secretion |
|  |  |  | physiological process |
|  |  |  | synaptic transmission |
|  |  |  | transmission of nerve impulse |
|  |  |  | transport |
|  |  |  |  |
| crooked legs (CG14938) | nucleus | ice binding | cell adhesion |
|  |  | nucleic acid binding | cell proliferation |
|  |  | RNA polymerase II transcription factor activity | cellular physiological process |
|  |  | transcription regulator activity | development |
|  |  | zinc ion binding | homoiothermy |
|  |  |  | mesoderm development |
|  |  |  | metabolism |
|  |  |  | morphogenesis |
|  |  |  | nucleobase, nucleoside, nucleotide and nucleic acid metabolism |
|  |  |  | physiological process |
|  |  |  | regulation of transcription from RNA polymerase II promoter |
|  |  |  | response to freezing |
|  |  |  | skeletal development |
|  |  |  | transcription |
|  |  |  | wing morphogenesis |
|  |  |  |  |
| Keap 1 (CG3962) |  | actin binding |  |
|  |  | binding |  |
|  |  | cytoskeletal protein binding |  |
|  |  | protein binding |  |
|  |  | structural constituent of cytoskeleton |  |
|  |  | structural molecule activity |  |
|  |  |  |  |
| CG5550 | extracellular matrix (sensu Metazoa) | binding | defense response to bacteria |
|  | extracellular region | receptor binding | physiological process |
|  |  | signal transducer activity | response to biotic stimulus |
|  |  |  | response to external stimulus |
|  |  |  |  |
| CG7142 |  | catalytic activity | catabolism |
|  |  | chymotrypsin activity | metabolism |
|  |  | hydrolase activity | physiological process |
|  |  | peptidase activity | protein metabolism |
|  |  | serine-type endopeptidase activity | proteolysis |
|  |  | trypsin activity |  |
|  |  |  |  |
| CG11099 | integral to membrane | rhodopsin-like receptor activity | G-protein coupled receptor protein signaling pathway |
|  |  |  |  |
| Technical knockout (CG7925) | cell | binding | behavior |
|  | cytoplasm | nucleic acid binding | biosynthesis |
|  | intracellular | structural constituent of ribosome | courtship behavior |
|  | mitochondrial ribosome | structural molecule activity | detection of mechanical stimulus during sensory perception of sound |
|  | mitochondrial small ribosomal subunit |  | male courtship behavior |
|  | mitochondrion |  | mechanosensory behavior |
|  | ribosome |  | metabolism |
|  |  |  | physiological process |
|  |  |  | protein biosynthesis |
|  |  |  | protein metabolism |
|  |  |  | response to abiotic stimulus |
|  |  |  | response to external stimulus |
|  |  |  | response to mechanical stimulus |
|  |  |  | sensory perception of sound |
|  |  |  |  |
| CG6859 | integral to peroxisomal membrane |  | cell organization and biogenesis |
|  |  |  | cellular physiological process |
|  |  |  | cytoplasm organization and biogenesis |
|  |  |  | organelle organization and biogenesis |
|  |  |  | peroxisome organization and biogenesis |
|  |  |  | physiological process |
|  |  |  | protein targeting to peroxisome |
|  |  |  | protein transport |
|  |  |  | transport |
|  |  |  |  |
| antennal protein 10 (CG6642) |  | binding | physiological process |
|  |  | carrier activity | response to external stimulus |
|  |  | odorant binding | sensory perception of chemical stimulus |
|  |  | pheromone binding |  |
|  |  | transporter activity |  |
|  |  |  |  |
| CG6231 | integral to membrane | carbohydrate transporter activity | cation transport |
|  |  | organic cation porter activity | cellular physiological process |
|  |  | transporter activity | extracellular transport |
|  |  |  | ion transport |
|  |  |  | physiological process |
|  |  |  | transport |
|  |  |  |  |
| Argonaute 1 (CG6671) | cell | binding | biosynthesis |
|  | cytoplasm | enzyme binding | development |
|  | eukaryotic translation initiation factor 2 complex | nucleic acid binding | metabolism |
|  | intracellular | translation factor activity, nucleic acid binding | miRNA-mediated gene silencing |
|  | RNA-induced silencing complex | translation initiation factor activity | miRNA-mediated gene silencing, mRNA cleavage |
|  |  | translation regulator activity | miRNA-mediated gene silencing, production of miRNAs |
|  |  |  | nucleobase, nucleoside, nucleotide and nucleic acid metabolism |
|  |  |  | physiological process |
|  |  |  | protein biosynthesis |
|  |  |  | protein metabolism |
|  |  |  | regulation of gene expression, epigenetic |
|  |  |  | RNA interference |
|  |  |  | RNA interference, production of siRNA |
|  |  |  | RNA interference, targeting of mRNA for destruction |
|  |  |  | synaptogenesis |
|  |  |  | translational initiation |
|  |  |  |  |
| gcm2 (CG3858) | cell | DNA binding | cell differentiation |
|  | intracellular | nucleic acid binding | cell proliferation |
|  | nucleus | transcription factor activity | cellular physiological process |
|  |  | transcription reg. activity | crystal cell differentiation |
|  |  |  | development |
|  |  |  | ectoderm development |
|  |  |  | embryonic development |
|  |  |  | embryonic plasmatocyte differentiation |
|  |  |  | glial cell differentiation |
|  |  |  | gliogenesis |
|  |  |  | morphogenesis |
|  |  |  | physiological process |
|  |  |  | plasmatocyte differentiation |
|  |  |  | regulation of transcription, DNA-dependent |
|  |  |  |  |
| CG8062 |  | monocarboylic acid transmembrane transporter activity |  |
|  |  |  |  |
| CG8258 | chaperonin-containing T-complex | ATPase activity, coupled | protein folding |
|  |  |  |  |
| CG9149 | cytosol | acetyl-CoA C-acetyltransferase activity |  |
|  |  |  |  |
| Cyp6a2 | membrane | electron carrier activity | response to caffeine |
|  | microsome |  | response to insecticide |
|  |  |  |  |
| Eip75B | nucleus | ligand-dependent nuclear receptor activity | antimicrobial humoral response |
|  |  | specific RNA pol.II transc. factor activity | ecdysis, chisin-based cuticle |
|  |  |  | ecdysone-mediated induction of salivary gland cell autophagic cell death |
|  |  |  | molding cycle, chitin-based cuticle |
|  |  |  | oogenesis (sensu Insecta) |
|  |  |  | regulation of ecdysteroid metabolic process |
|  |  |  | regulation of transc., DNA-dependent |
|  |  |  |  |
| fusilli |  | mRNA binding | epidermal growth factor receptor signaling pathway |
|  |  |  |  |
| Glycogenin |  | glycodenin glucosyltransferase activity | mesoderm development |
|  |  |  |  |
| Gmd |  | GDP-mannose 4,6-dehydratase activity | ‘de novo’ GDP-L-fucose biosynthetic process |
|  |  |  | GDP-L-fucose biosynthetic process |
|  |  |  |  |
| icarus |  | small GTPase regulator activity | apposition of dorsal and ventral imaginal disc-derived wing surfaces |
|  |  |  | negative regulation of JNK activity |
|  |  |  | negative regulation of JNK cascade |
|  |  |  |  |
| mRpL3 | mitochondrial large ribosomal subunit | structural constituent of ribosome | translation |
|  | mitochondrial small ribosomal subunit |  |  |
|  |  |  |  |
| Pp2B-14D | calcineurin complex | calcium-dependent protein serine/threonine phosphatase activity | female meiosis |
|  |  | calmodulin binding | protein amino acide dephosphorylation |
|  |  | protein binding | wing disc development |
|  |  | protein serine/threonine phosphatase activity |  |
|  |  |  |  |
| real-time | mitochondrion | phosphatidylinositol transporter activity |  |
|  |  |  |  |
| Rgk1 |  | GTPase activity |  |
|  |  |  |  |
| RpA-70 | DNA replication factor A comlex | single-stranded DNA binding | DNA-dependent DNA replication |
|  | nucleus |  |  |
|  |  |  |  |
| Su(var)205 | centric heterochromatin | chromatin binding | chromatin silencing at centromere |
|  | chromosome, pericentric region | histone binding | chromatin silencing |
|  | chromosome, telomeric region | methylated histone residue binding | chromosome organization and biogenesis |
|  | condensed chromosome | mRNA binding | establishment of chromatin silencing |
|  | condensed nuclear chromosome, pericentric region | rDNA binding | negative regulation of transcription, DNA-dependent |
|  | heterochromatin | satellite DNA binding | positive regulation of transcription, DNA-dependent |
|  | nuclear heterochromatin | transcription activator activity |  |
|  | nucleus | transcription repressor activity |  |
|  | polytene chromosome chromocenter |  |  |
|  | polytene chromosome puff |  |  |
|  | polytene chromosome, telomeric region |  |  |
|  |  |  |  |
| Cyp4p2(CG1944) | Listed in Table S4 |  |  |
| Gclc (CG2259) | Listed in Table S4 |  |  |
| CG9842 | Listed in Table S4 |  |  |
| Blistery(CG9379) | Listed in Table S4 |  |  |
