# Supplementary material for: Adaptive Gene Expression Divergence Inferred from Population Genomics
Source: PLoS Genet. 2007 Oct 26;3(10):e187. doi: 10.1371/journal.pgen.0030187 (PMC2042001; doi:10.1371/journal.pgen.0030187)
Supplement: Table S6 — (192 KB DOC) [file pgen.0030187.st006.doc]

Table S6. Gene Ontology information for gene with increases in expression and evidence for recent adaptive evolution in the coding region.

| Gene | Cellular Component | | Molecular Function | | Biological Process | |
| --- | --- | --- | --- | --- | --- | --- |
| Roc1a (CG16982) | GO:0005623 | cell | GO:0005488 | binding | GO:0009056 | catabolism |
|  | GO:0005737 | cytoplasm | GO:0003824 | catalytic activity | GO:0007154 | cell communication |
|  | GO:0005622 | intracellular | GO:0005515 | protein binding | GO:0008283 | cell proliferation |
|  | GO:0005634 | nucleus | GO:0004842 | ubiquitin-protein ligase activity | GO:0050875 | cellular physiological process |
|  | GO:0019005 | SCF ubiquitin ligase complex | GO:0008270 | zinc ion binding | GO:0008152 | metabolism |
|  |  |  |  |  | GO:0007582 | physiological process |
|  |  |  |  |  | GO:0019538 | protein metabolism |
|  |  |  |  |  | GO:0006464 | protein modification |
|  |  |  |  |  | GO:0016567 | protein ubiquitination |
|  |  |  |  |  | GO:0042787 | protein ubiquitination during ubiquitin-dependent protein catabolism |
|  |  |  |  |  | GO:0006508 | proteolysis |
|  |  |  |  |  | GO:0007165 | signal transduction |
|  |  |  |  |  | GO:0007224 | smoothened signaling pathway |
|  |  |  |  |  | GO:0006512 | ubiquitin cycle |
|  |  |  |  |  |  |  |
| CG17856 | GO:0005623 | cell | GO:0003824 | catalytic activity | GO:0006118 | electron transport |
| electron trans | GO:0005737 | cytoplasm | GO:0005215 | transporter activity | GO:0008152 | metabolism |
|  | GO:0005622 | intracellular | GO:0008121 | ubiquinol-cytochrome-c reductase activity | GO:0006122 | mitochondrial electron transport, ubiquinol to cytochrome c |
|  | GO:0005739 | mitochondrion |  |  | GO:0007582 | physiological process |
|  | GO:0005750 | respiratory chain complex III (sensu Eukaryota) |  |  |  |  |
|  |  |  |  |  |  |  |
| Frequenin 2 (CG5907) | GO:0005623 | cell | GO:0005488 | binding | GO:0019722 | calcium-mediated signaling |
|  | GO:0005737 | cytoplasm | GO:0005509 | calcium ion binding | GO:0007154 | cell communication |
|  | GO:0016023 | cytoplasmic membrane-bound vesicle | GO:0005516 | calmodulin binding | GO:0007267 | cell-cell signaling |
|  |  |  |  |  |  |  |
|  |  |  |  |  |  |  |
|  |  |  |  |  |  |  |
| Table S6 continued. | | | | | | |
| Gene | Cellular Component | | Molecular Function | | Biological Process | |
| Frequenin 2  cont. | GO:0005578 | extracellular matrix (sensu Metazoa) | GO:0030234 | enzyme regulator activity | GO:0050875 | cellular physiological process |
|  | GO:0005576 | extracellular region | GO:0005515 | protein binding | GO:0007269 | neurotransmitter secretion |
|  | GO:0005622 | intracellular |  |  | GO:0007582 | physiological process |
|  | GO:0008021 | synaptic vesicle |  |  | GO:0009605 | response to external stimulus |
|  |  |  |  |  | GO:0007165 | signal transduction |
|  |  |  |  |  | GO:0006810 | transport |
|  |  |  |  |  | GO:0016192 | vesicle-mediated transport |
|  |  |  |  |  |  |  |
| 1.28 (CG9397) | GO:0016469 | proton-transporting two-sector ATPase complex | GO:0005524 | ATP binding | GO:0015986 | ATP synthesis coupled proton transport |
|  |  |  | GO:0046933 | hydrogen-transporting ATP synthase activity, rotational mechanism | GO:0007275 | development |
|  |  |  | GO:0046961 | hydrogen-transporting ATPase activity, rotational mechanism | GO:0009790 | embryonic development |
|  |  |  |  |  | GO:0007382 | specification of segmental identity, maxillary segment |
|  |  |  |  |  |  |  |
| Scylla (CG7590) |  |  |  |  | GO:0045926 | negative regulation of growth |
|  |  |  |  |  |  |  |
| CG10418 | GO:0005623 | cell | GO:0005488 | binding | GO:0008152 | metabolism |
|  | GO:0005622 | intracellular | GO:0003676 | nucleic acid binding | GO:0000398 | nuclear mRNA splicing, via spliceosome |
|  | GO:0005634 | nucleus | GO:0003723 | RNA binding | GO:0006139 | nucleobase, nucleoside, nucleotide and nucleic acid metabolism |
|  | GO:0030532 | snRNP complex |  |  | GO:0007582 | physiological process |
|  | GO:0005732 | small nucleolar RNP complex |  |  | GO:0000381 | regulation of alternative nuclear mRNA splicing, via spliceosome |
|  | GO:0005688 | snRNP U6 |  |  |  |  |
|  | GO:0005681 | spliceosome complex |  |  |  |  |
|  |  |  |  |  |  |  |
| CG11249 |  |  | GO:0004743 | pyruvate kinase activity | GO:0006096 | glycolysis |
|  |  |  |  |  |  |  |
| Table S6 continued. | | | | | | |
| Gene | Cellular Component | | Molecular Function | | Biological Process | |
| Virilizer (CG3496) | GO:0005623 | cell | GO:0005488 | binding | GO:0007275 | development |
|  | GO:0016021 | integral to membrane | GO:0003676 | nucleic acid binding | GO:0007549 | dosage compensation |
|  | GO:0005622 | intracellular |  |  | GO:0008152 | metabolism |
|  | GO:0005634 | nucleus |  |  | GO:0006139 | nucleobase, nucleoside, nucleotide and nucleic acid metabolism |
|  |  |  |  |  | GO:0007582 | physiological process |
|  |  |  |  |  | GO:0007539 | primary sex determination, soma |
|  |  |  |  |  | GO:0040029 | regulation of gene expression, epigenetic |
|  |  |  |  |  | GO:0048024 | regulation of nuclear mRNA splicing, via spliceosome |
|  |  |  |  |  | GO:0007530 | sex determination |
|  |  |  |  |  |  |  |
| Tetraspanin 42 Ec (CG12847) | GO:0005623 | cell |  |  |  |  |
|  | GO:0016021 | integral to membrane |  |  |  |  |
|  |  |  |  |  |  |  |
| CG10418 | GO:0005623 | cell | GO:0005488 | binding | GO:0008152 | metabolism |
| nuclear splicing | GO:0005622 | intracellular | GO:0003676 | nucleic acid binding | GO:0000398 | nuclear mRNA splicing, via spliceosome |
|  | GO:0005634 | nucleus | GO:0003723 | RNA binding | GO:0006139 | nucleobase, nucleoside, nucleotide and nucleic acid metabolism |
|  | GO:0030532 | small nuclear ribonucleoprotein complex |  |  | GO:0007582 | physiological process |
|  | GO:0005732 | small nucleolar ribonucleoprotein complex |  |  | GO:0000381 | regulation of alternative nuclear mRNA splicing, via spliceosome |
|  | GO:0005688 | snRNP U6 |  |  |  |  |
|  | GO:0005681 | spliceosome complex |  |  |  |  |
